# Lymphovenous anastomosis: microsurgical innovation and clinical outcomes in breast cancer-related lymphedema care

**DOI:** 10.3389/fsurg.2025.1731257

**Published:** 2026-01-12

**Authors:** Claire J. Lee, Eric S. Hong, Dana J. Rhee, Dongwon Choi

**Affiliations:** 1 La Cañada High School, La Cañada, CA, United States; 2 Department of Surgery, Beth Israel Deaconess Medical Center, Boston, MA, United States; 3 Lexington High School, Lexington, MA, United States

**Keywords:** breast cancer-related lymphedema (BCRL), lymphatic microsurgical preventive healing approach (LYMPHA), lymphedema care, lymphovenous anastomosis (LVA), vascularized lymph node transfer (VLNT)

## Abstract

Lymphovenous anastomosis (LVA) has emerged as an important physiologic microsurgical procedure for patients with breast cancer–related lymphedema (BCRL) with the goal of restoring lymphatic drainage rather than providing just palliative care for symptoms of swelling. A multicenter randomized controlled trial (RCT) in 2024 (N-LVA) found improvements in the Lymph-ICF physical and mental function domains, and decreased use of compression garments, despite modest changes in total quality of life (QoL) and limb volume at 6 months. Meta-analyses have found average reductions of 30%–35% in excess limb size and nearly two fewer cellulitis episodes per year after LVA and vascularized lymph node transfer (VLNT). As the surgical technology continues to improve (e.g., prophylactic LYMPHA procedures, high-resolution lymphatic imaging, robotic supermicrosurgery) and as LVA becomes more widely adopted within experienced surgical centers, precision surgery will be increasingly considered in lymphedema care. Collectively, these advancements represent a movement toward physiologic reconstruction in lymphedema care and the next initiatives will focus on patient selection and eligibility optimization, state-of-the-art surgical technology optimization, and standardizing outcome measures to achieve sustained improvements in QoL.

## Introduction

BCRL is a chronic arm (occasionally chest/breast) swelling that results as a side effect of breast cancer treatment, especially axillary lymph node dissection and radiation of the axillary nodal basin following lymph node removal (1, 2). It is a significant survivorship problem for a very large global population of breast cancer survivors (3). The rate of BCRL has presented varying results in the literature. The cumulative incidence has been reported to range from 5% to over 40%, depending on several factors including the type of surgical procedure, use of combination therapy, and the criteria used to evaluate the patient for BCRL (3–5). Additional contemporary cohort studies also reflect this variability (1, 6–9).

The most frequently used clinical definition of BCRL has historically involved an increase in limb circumference of at least 2 cm or a relative arm volume change of 10% or more compared with the contralateral arm (4, 10–12). Prospective evidence indicates that approximately 20%–30% of women undergoing axillary lymph node dissection (ALND) will develop a degree of lymphedema within a few years, while sentinel lymph node biopsy by itself carries a lower risk, often less than 10% (1, 4, 6, 8). The most significant risks include elevated BMI at the time of diagnosis, the number of lymph nodes taken out, regional node radiotherapy, and the development of a postoperative infection like cellulitis (4, 6, 9, 13–15).

Over time, lymphedema may cause skin changes such as fibrosis and hyperkeratosis (16). Patients with BCRL may experience anxiety, depression, and social avoidance (17). The swollen limb may interfere with daily activities, restrict clothing choices, and burden patients economically due to the treatment costs and lost wages (18). In severe, long-standing cases, complications such as lymphorrhea (leakage of lymphatic fluid), nonhealing ulcers, or even a heightened threat of lymphangiosarcoma (Stewart-Treves syndrome) may develop (16, 19).

In BCRL, cancer therapies, particularly ALND and radiotherapy, damage or disrupt axillary lymphatic vessels and nodes, which impairs arm lymph drainage (5, 16, 20). The remaining lymphatics have limited capacity to handle the normal volume of lymph fluid and thus develop a buildup of protein-rich lymph within the arm's interstitial tissues. Eventually, this stasis of the fluid produces a cascade of pathophysiologic responses: adipose infiltration of tissues, subcutaneous tissue fibrosis, and damage to lymphatic function (20–22). If the lymphatic insufficiency persists, edema becomes visible (Stage I), which is usually soft and pitting and tends to improve when the limb is elevated. Without treatment, the chronic inflammation results in remodeling of the tissues (Stage II), where the edema fails to pit easily and fibrotic reactions cause the edema to be harder and non-resolving even on elevation (12, 16, 22). In more advanced Stage III, referred to as lymphostatic elephantiasis, significant tissue fibrosis, fat infiltration, and skin changes such as papillomas and hyperkeratosis appear. At this point, the condition is considered irreversible, and only partial improvement is possible with treatment (12, 22, 23).

## Traditional management strategies for lymphedema

For decades, the mainstay of lymphedema management has been non-surgical, based on Complete Decongestive Therapy (CDT) (24). CDT consists of compression, manual lymphatic drainage (MLD), skincare, and appropriate physical activity. Access to certified lymphedema therapists for the application of MLD may be limited outside of a specialty clinic, and the treatment is time-consuming and expensive without adequate insurance (25–27).

More recently, there has been greater development of microsurgical, physiologic procedures to restore lymphatic function rather than simply excising tissues (28). Two categories of surgical procedures are recognized: (1) ablative or debulking procedures such as liposuction (suction-assisted lipectomy) or the Charles procedure, which reduce limb volume by removing fluid and solid tissue (12, 28, 29); and (2) physiologic procedures such as lymphaticovenous anastomosis (bridging lymphatics to veins) or VLNT, which aim to reestablish lymphatic drainage (29, 30).

Dating back to the late 20th century and gaining traction in the 2000s, VLNT consists of the harvesting of lymph nodes from one part of the body, and transplanting them into the lymphedematous limb. Common donor sites are the groin (inguinal nodes), neck (supraclavicular nodes) or abdomen (as an omental flap or contralateral axillary nodes). To reduce donor-site complications, techniques such as reverse lymphatic mapping are used to preserve native drainage pathways and reduce the risk of iatrogenic lymphedema (31).

The transplanted nodes are anastomosed to axillary or elbow region blood vessels of the involved arm, and over the course of a few months, they may function as a “lymphatic pump” or a nexus to absorb lymph fluid and then facilitate drainage of lymph fluid into the venous circulation through new lymphatic or lymphovenous connections. VLNT has resulted in limb volume reduction and clinical improvement, although the precise mechanisms by which this occurs are under investigation; it is thought that transplanted nodes promote lymphangiogenesis and directly absorb interstitial fluid (32).

The development of lymphedema surgery has progressed from strictly reductive measures towards microsurgical lymphatic pathway reconstruction, which led to physiologic procedures like LVA and VLNT. The following section examines LVA, its methods and the most recent evidence from clinical series.

## Lymphovenous anastomosis (LVA): principles and technique

LVA, also referred to as lymphovenous bypass, is a supermicrosurgical procedure that directly connects lymphatic vessels with the venous system. In an LVA, the suture of one or more of the limb's lymphatic collecting vessels to adjacent small veins (venules, typically <1 mm in diameter) establishes a shunt through which lymph enters the vein (33). The connections are most commonly made within the distal limb (forearm or hand for upper limb BCRL) where lymphatic channels are most likely to be present and viable, and where venules are accessible in the subcutaneous plane (34). The procedure may be done under local anesthesia or general anesthesia, at the preference of the surgeon and the patient. Small incisions, typically 2–3 cm or smaller, are made on the arm, and the surgeon uses the operating microscope to identify the lymphatic vessels and the venules (35). Where to make the incisions is guided by preoperative indocyanine green (ICG) lymphography: ICG injected intradermally into the hand helps identify where lymphatic channels run, and they may be visualized on a near-infrared camera as “linear” patterns of functional lymphatics and “dermal backflow” patterns where the lymph stagnates (36, 37).

Anastomoses are performed under high magnification using a microscope, typically 20–30× with supermicrosurgical instruments and 11-0 or 12-0 nylon sutures (38). The lymphatic vessels used for LVA are approximately 0.3–0.8 mm in diameter, threadlike, and translucent, which makes the use of dye helpful for identification during surgery (39, 40). An interdigital injection of a blue dye may facilitate visualization of the lymphatics on the operative site.

There are two patterns for the anastomosis: a common procedure involves end-to-end (lymphatic cut end being connected to a venule cut end) or end-to-side (lymphatic inserted into a small incision on the side of a vein) (41). Surgeons ensure that the lymphatic fluid will flow into the vein and never the reverse (42). Venous pressure tends to be low usually in these small veins, particularly on an elevated limb; thus, provided the anastomosis is performed properly, the lymph should drain into the vein (33). Some surgeons take particular care regarding the choice of a vein where there's zero reflux and prefer the end-to-side type of configuration where there's less potential for the venous blood back flowing into the lymphatic (43).

Multiple LVAs are typically performed on a single limb such as this, typically 2–4 anastomoses, sometimes more, for maximum drainage of the lymph. In a series of 100 cases of LVA operation, the mean number of LVAs performed on each patient by the initial surgery was approximately 3.6, with some requiring additional procedures during later stages (44).

**Figure 1:**
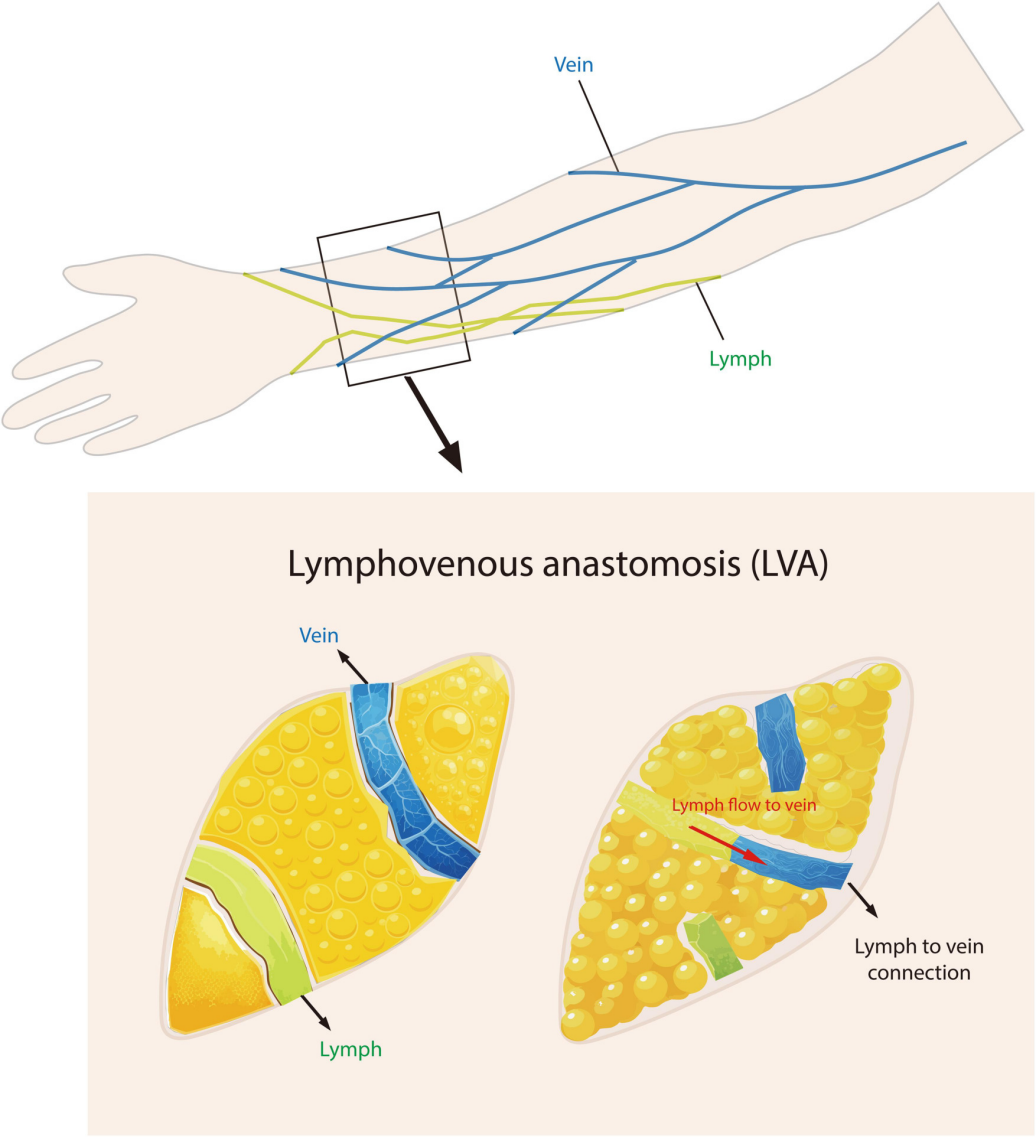
Schematic illustration of lymphovenous anastomosis (LVA). Top: superficial lymphatic collectors (green) and companion veins (blue) in the upper arm area, where LVA surgery is most commonly performed. Bottom left: identification of a functional lymphatic vessel adjacent to a venule located in the subcutaneous area. Bottom right: creation of a connection between the two systems, with arrows indicating lymph fluid flowing from the lymphatic vessels into the vein.

Successful LVA creates a new drainage path for lymph. The patient may experience softer edema and less heaviness relatively shortly after the surgery, sometimes within days, as excess fluid has a new route of escape (45). LVA typically performs optimally in earlier stages of lymphedema, where the lymphatic pathways have not entirely obliterated (46, 47). There may be a scarcity of usable lymphatics and severe fibrosis [International Society of Lymphology (ISL) Stage III], where the very small anastomoses may get flattened due to the elevated tissue pressure and make LVA ineffectual. Another limitation is that the chronic fatty deposits common in longstanding lymphedema are not eliminated by LVA; therefore, even when fluid drainage improves, the limb girth attributable to fat remains (48). An overview of the anatomic relationships and the microsurgical principles of the LVA procedure is shown in Figure 1.

## Indications, patient selection, and intraoperative considerations

Selecting appropriate patients is important for the success of LVA. The ideal candidate is one with early or moderate BCRL (ISL Stage I or II) who has a patent lymphatic channel as determined on imaging and who has not acquired extensive irreversible changes (46, 49). These cases typically represent the first few years of lymphedema when edema remains measurable and symptomatic despite conservative therapy. The situation is different for patients who have advanced Stage II or Stage III (hard fibrous edema, massive girth discrepancy, skin changes of a papillomatous type). For these cases, the benefit of LVA alone is limited, and combined or alternative surgeries such as the addition of VLNT or a debulking procedure may provide greater benefit (49).

### Preoperative assessment

Before undergoing the LVA procedure, imaging tests are usually performed to evaluate functional lymphatics (36, 50, 51). The most common form of imaging is ICG lymphography. A small amount of ICG dye is administered intradermally into the hand, and the lymphatics are then visualized in real-time with an infrared camera (51). Depending on the results, a positive finding might show a dermal backflow pattern in the proximal arm while having intact linear channels in the distal arm, suggesting there are functioning lymphatics that can be accessed and diverted into upstream veins (36, 51).

Lymphoscintigraphy (nuclear scan) provides an objective assessment of lymphatic function and typically shows delayed or absent uptake of the tracer in the axilla in patients diagnosed with BCRL, but does not provide as much level of detail in mapping individual lymphatics (23, 52). Duplex ultrasound may be performed for confirmation of venous patency and localization of target veins. Any active infections such as cellulitis should be fully treated and resolved before surgery.

### Intraoperative considerations

Some surgeons begin by injecting patent blue dye into the web spaces of the hand before surgery. This dye travels along lymphatics, stains the lymphatics light blue and makes them visible under the microscope. The surgeon makes small incisions where preoperative mapping has shown suitable lymphatic vessels (53). They then identify a lymphatic, often a wisp of material or a clear thread, sometimes a bead of lymph fluid visible most prominently, and a nearby venule, a small vein that typically accompanies arteries or courses in superficial fat. The surgeon places several fine sutures (typically 2–4) to connect the lymphatic and venule, most often in an end-to-end manner to ensure proper alignment (53, 54).

### Directional concern

Lymphatic and venous channels contain unidirectional valves. The surgeon must perform a lymphatic anastomosis downstream of any working valves in the vein to ensure unidirectional flow from the lymphatic to the vein (42). Other surgeons may create venous supercharging, an additional venous outflow or anastomosis to reduce venous pressure and backflow into the lymphatics (43). This issue is less relevant in low-pressure venules.

## Clinical outcomes of LVA in BCRL

The clinical results of LVA for BCRL have been reported in multiple clinical series for the past 20 years, as well as more recent comparative studies (46, 47, 55, 56). The evidence indicates that LVA may reduce limb volume, relieve symptoms and improve daily function for many individuals, especially those with early-stage lymphedema (55, 57). The following section summarizes the main results of recent studies on LVA outcomes.

### Volume and circumference reduction

Most studies reported a decrease in either the volume or circumference of the affected arm after LVA was performed (55). Cornelissen et al. analyzed 15 studies of LVA for BCRL in a systematic review. 13 of these studies showed either a decrease in volume or limb circumference after the surgical intervention of LVA and 12 studies showed improvement in limb-related symptoms or QoL in 50%–100% of patients in their studies (46). The magnitude of volume reduction reported by these studies varied widely, from minor improvements to greater than a 50% reduction in excess volume. The authors stated that LVA was beneficial for early-stage BCRL, but emphasized the need for stronger evidence and standardized outcome measures.

A commonly referenced case series is from Chang et al., whose 2013 prospective trial included reporting on 100 LVA cases (predominantly for BCRL) (44). In their study group of patients with upper extremity BCRL, there was a reported decrease in average excess limb volume from 32% to 18% postoperatively, representing about 42% less swelling on average at 1 year post-operation. Patients in stage I and stage II achieved much more size reductions, approximately a 60% decrease in excess limb volume. Conversely, patients with stage III disease only achieved a reduction of approximately 15%.

### Functional and quality of life improvement

In addition to objective measures of volume decrease, multiple studies have reported that patient-reported outcomes improved after LVA. Patients often reported that the arm felt lighter, less painful, and “more normal” (58, 59). Patients also showed an improvement in range of motion due to decreased stiffness. QoL in lymphedema may be quantified using tools such as the Lymph-ICF questionnaire, LYMQOL, and LYMPH-Q (56, 58–60).

In a recent series of 100 consecutive patients followed for 2 years after LVA, 84% showed an improved Lymph-ICF score, therefore better QoL/function, and more than half of the patients improved 10 points (59). Additionally, 43% of the patients completely stopped wearing compression garments, and 18% only wore them occasionally, which showed an improvement in QoL. These findings were supported by a 2024 RCT of 92 patients with early-stage BCRL, in which LVA produced sustained improvements in both physical and mental functioning on the Lymph-ICF compared with conservative therapy alone. Notably, 42% of LVA patients partially or completely discontinued compression garments, compared with none in the control group (56).

### Infection reduction

For many patients, the debilitating recurrence of cellulitis is a part of lymphedema (61). LVA may significantly decrease the risk of acute infection (62). The meta-analysis conducted by Meuli et al. gathered the studies and showed that the annual number of occurrences of cellulitis subsequent to the microsurgical procedures (LVA or VLNT) decreased on average by about 1.9 (57). Patients with multiple recurrent infections typically experience a decrease in the number of infections after lymphedema surgery has been completed (63).

### Objective lymphatic function

Wolfs et al. examined the patency of LVAs at 12 months postoperatively using ultrasound and indocyanine green (ICG) lymphography and reported 76% of patients maintained at least one patent anastomosis at 1 year. Patients with patent LVAs had greater volume reduction and improved quality of life compared with those whose anastomoses were occluded (64). This supports the notion that patency of LVAs is important and calls for methods, or postoperative care that maintain the patency of the anastomoses. For example, several have proposed that continued use of the arm, including muscle contractions, keep the flow going through the new channels.

### Comparative studies and trials

Previously, the bulk of LVA effectiveness evidence was based on single-arm case series without a comparison group. More recent studies are yielding higher levels of evidence. One development is the first large, well-designed RCT of LVA (56). Early in 2024, results of a multicenter RCT (the “N-LVA” trial) 6-month interim analysis were published in Scientific Reports (56).

In this trial, patients with initial-stage BCRL were randomized to receive LVA surgery and standard treatment vs. standard treatment (CDT) alone, and outcomes were assessed at 3 and 6 months. The 6-month results were nuanced: the LVA group demonstrated significant improvement on certain aspects of the Lymph-ICF QoL questionnaire (physical function and mental function) over baseline, while the CDT-only group did not. Yet, the overall Lymph-ICF scores (composite total) did not differ significantly between groups by 6 months, perhaps because changes take longer, or the sample remains small.

What was importantly noted was that the arm volumes had not decreased significantly in the two groups by 6 months: the LVA group had a trend towards reduction (∼20 mL average volume drop), albeit not significant, and the control group also had a small and not significant decline. One of the surprise results was that 41% of the LVA group were able to stop wearing compression garment(s) at least part-time by 6 months, while 0% of the conservative group could. That aligns with the reports of LVA being able to make garment dependency decline quite rapidly (59, 65).

The trial will go on to a 12-month and longer-term analysis when more distinction may be demonstrated (particularly since benefits from LVA may accrue after 6 months as changing tissues revert). However, this RCT offers significant proof that LVA can provide QoL benefits early on, even if volume changes do not show immediately. It also shows that a proportion of the group (almost half) improve enough to drop compression, a measurable victory for survivors.

A randomized first-in-human pilot in 2020 by van Mulken et al. has shown feasibility and safety when performing robot-assisted LVA (with the MUSA robot). The upper extremity lymphedema (UEL) index and QoL were both improved in robotic and manual groups, with significantly reduced anastomosis time through the learning curve (33–16 min). Manual LVA was found to have slightly superior microsurgical performance scores (66).

To date, no randomized trial of LVA vs. another type of surgery (e.g., VLNT) or true placebo surgery has been completed (67). The N-LVA trial described above is basically LVA vs. no LVA (56). The consensus of numerous systematic reviews is that although LVA benefits are evident in many studies, the quality of the evidence remains confounded by heterogeneity and a lack of blinding or control in most reports.

There was a substantial systematic review and meta-analysis of 150 studies involving more than 6,000 microsurgery-treated patients by Meuli et al. in 2023 (57). The meta-analysis calculated a 35.6% reduction of excess circumference and a 32.7% reduction of excess volume after treatment (primarily LVA and VLNT). These aggregate results confirm that microsurgery can virtually eliminate one-third to one-half of the lymphedema burden for many cases. Furthermore, that meta-analysis reaffirmed the substantial reduction of the previously noted infection rates and demonstrates the health benefits and not just tape-measure numbers.

### Comparative effectiveness of LVA vs. VLNT

Although both LVA and VLNT improve lymphedema, their effects may differ in timing and magnitude. Kappos et al. reported a 24-month retrospective cohort study of 70 patients undergoing VLNT and 42 patients undergoing LVA. LVA resulted in a quick early effect with a relative reduction in circumference of 7.15% at 3 months, but this declined to 2.04% by 6 months. VLNT was slower with a peak at 3 months (3.32%) and a second peak at 18 months (3.29%). Relative excess volume reductions also favored LVA early (134.5% improvement relative to baseline) but diminished over time. By 24 months, reduction rates were similar (VLNT: 1.88%; LVA: 1.84%). Nonetheless, LVA did not incur complications while the complication rate in VLNT was 14.3%, in line with reported ranges of 15%–20% in studies (47).

Both were effective, but not universally better than the other; they have “different patterns of improvement over time” (47). This information could be used to individualize patient counseling. For example, a sports enthusiast who wants to get back to training may favor LVA for the rapid resolution, while a patient with severe disease may prefer a slower resolution initially by VLNT, if that had the potential for more reduction over time.

Table 1 summarizes major clinical trials and studies of LVA for BCRL, which describe their general findings and methodologies. The preponderance of evidence from recent studies informs us of several things: (1) Outcomes show a broad range indicating that some patients are “super-responders” and some patients have little or no degrees of improvement, likely in part from the disease burden and the success of the procedure being performed; (2) Strong comparative evidence is just starting to develop (47).

**Table 1:** Summary of key clinical studies evaluating LVA for breast cancer–related lymphedema (BCRL).

Study (Year)	Design/Patients	Follow-up	Outcomes
Cornelissen et al. (2018) (46)	Systematic review of 15 LVA studies in BCRL patients (*n* = 263)	Average ∼20 months	13 of 15 studies reported volume or circumference reduction. 12 studies reported symptom or QoL improvement (range: 50%–100%). LVA most effective in early stage BCRL.
Chang et al. (2013) (44)	Prospective cohort; 100 consecutive LVA cases (mostly BCRL)	12 months	Mean excess limb volume decreased from 32% (pre-op) to 18% (post-op), a 42% reduction. Stage I–II patients showed the greatest improvement (60% reduction), while Stage III showed limited response (15%). Supports early intervention for optimal results.
Chang et al. (2023) (48)	Prospective cohort; 158 patients with BCRL treated by staged liposuction and then LVA	Median 30 months (range 23–36)	Median arm volume excess decreased from 838.3 mL (pre-op) to 7.8 mL (7 days) and 43.7 mL (follow-up). Erysipelas episodes significantly declined; 6.3% were garment-free for ≥6 months. No serious complication.
Jonis et al. (2024) (56)	Randomized controlled trial; 92 patients with unilateral BCRL (ISL stage 1–2a)	6 months (interim analysis)	Physical and mental function domains of the Lymph-ICF questionnaire were significantly different in LVA vs. CDT. No difference between groups in total Lymph-ICF score or volume of the affected limb. UEL index reduced at 3 months in LVA group but not at 6 months. 42% of patients in LVA group discontinued or reduced use of compression garments vs. 0% in CDT group. Adverse event rate was similar in both groups, with one reported SAE in the LVA group.
Meuli et al. (2023) (57)	Systematic review and meta-analysis; 150 studies, 6,496 patients (including BCRL)	Mean 20.4 months (range across studies)	Pooled meta-analysis indicated a reduction in excess circumference by −35.6% (29 studies, *n* = 1,002), a reduction in excess volume by −32.7% (12 studies, *n* = 587), and a reduction of −1.9 episodes/year in cutaneous infections (8 studies, *n* = 248). Qualitative synthesis found improvement in quality of life in 22% of studies, reduction in use of compression garments in 19%, and patient satisfaction in 21%. Substantial heterogeneity in outcome measures was reported, drawing attention to the need for standardized staging and reporting.
Kappos et al. (2025) (47)	Retrospective cohort study; 112 patients with chronic BCRL treated with either VLNT (*n* = 70) or LVA (*n* = 42)	24 months	Both VLNT and LVA reduced arm circumference significantly. LVA resulted in a peak effect early (7.15% relative reduction at 3 months), followed by a reduction by 6 months (2.04%) and stabilizing. VLNT was characterized by a gradual pattern with two peak responses at 3 (3.32%) and 18 (3.29%) months with sustained superior long-term efficacy (1.88% at 24 months vs. 1.84% for LVA). Relative excess reduction also peaked early (VLNT: 95.8%, LVA: 134.5%) with declining effect at 24 months. VLNT also exhibited greater complication (14.3%) than LVA (0%). Frequency of manual lymphatic drainage was reduced in both groups but to a much greater extent in LVA patients. Results indicate LVA has early advantages while VLNT has more long-term improvement.
Boccardo et al. (2014) (76)	Prospective series; 74 breast cancer patients undergoing dissection of the axilla + LYMPHA (preventive lymphaticovenous anastomosis); 71 follow-up completed	Mean follow-up of 4 years (1, 3, 6, 12 months and then annually)	Lymphedema rate of 4.05% (3/74); follow-up lymphoscintigraphy showed long-term patency of anastomoses; considerable decrease in lymphatic transport index compared to pre-op values.
van Mulken et al. (2020) (66)	First-in-human randomized pilot study; 20 women with BCRL were randomized to robot-assisted (MUSA) or manual LVA	3 months	Robotic LVA was safe and feasible. All anastomoses were patent. QoL and UEL index were improved in both groups. Robot group demonstrated a steep learning curve (the time to anastomose decreased from 33 to 16 min). Manual group performed superior microsurgical scores (SAMS/UWOMSA).

The lack of randomized studies has provided criticism of the procedure, but now that interim RCT results and comparative cohorts are available, the medical community is increasingly comfortable with the role of LVA (56). The current consensus, based on recent expert panel recommendations and systematic reviews, is that physiologic procedures such as LVA and VLNT are significant for the treatment of BCRL when conservative therapies fail, and should be considered before debulking approaches (15, 46, 49, 57). Even with surgery though, it should be noted that there is still no cure for lymphedema and most patients will not return to their pre-cancer state (58).

## Complications and limitations of LVA (and other surgical approaches)

One of the advantages of LVA is its low complication rate. Unlike most major surgeries, LVA does not generally cause serious complications when performed by an experienced microsurgeon (68). The procedure does not create a new lymphatic deficit (no donor site that could develop lymphedema) and does not involve large-vessel anastomoses that could lead to limb-threatening complications (49). LVA is a precise and superficial procedure and poses minimal risk of injury to major vessels or nerves.

In contrast, Ablative procedures, such as the Charles procedure and massive liposuction, have elevated risk profiles of complications (69). The Charles procedure is a radical excision technique that carries risk for complications related to wound healing, large scars, and increased length of hospitalization (28). The procedure effectively trades lymphedema for extensive scarring and is reserved for extreme cases (70).

Suction-assisted lipectomy made popular in Sweden for lymphedema has a relatively safe profile like cosmetic liposuction, but requires lifelong adherence to compression therapy afterward, and it predominantly targets fat, not fluid (63, 71). Liposuction can cause hematoma and contour deformity, or infection, but rates are small when done properly. In one large series of liposuction for arm lymphedema there were minimal significant complications, such as temporary sensory change and mild edema (72).

### Limitations of LVA

The main limitations involve the selection of patients and the amount of benefit that can be expected from the procedure. LVA requires that functional lymphatics are present, therefore patients without evidence of an intact lymphatic system are not good candidates for LVA (51). For example, a patient who had extensive axillary surgery and/or radiation decades ago, and is now presenting with a fibrotic and indurated arm, may not have sufficient lymphatic channels, therefore VLNT or debulking procedure may be more appropriate (73). Furthermore, LVA outcomes are operator-dependent, as the procedure is technically difficult and has a long learning curve (43).

Patients may report improved symptoms despite little change in limb volume. This could be due to the exchange of fluid for adipose tissue. Others may report a measurable change in volume but continue to have pain (58, 59). It also needs to be emphasized that LVA does not provide an immediate solution and patients should maintain realistic expectations (57).

Table 2 provides a comparative summary of LVA and VLNT, which are often performed in a similar patient population. LVA generally offers an effective, less invasive solution with faster results, while VLNT under ideal conditions may provide longer sustained improvement, but it does require additional surgery and risk to the donor site (47, 57).

**Table 2:** Comparison of lymphovenous anastomosis (LVA) and vascularized lymph node transfer (VLNT) for breast cancer-related lymphedema (BCRL).

Aspect	Lymphovenous anastomosis (LVA)	Vascularized lymph node transfer (VLNT)	References
Approach	Supermicrosurgical bypass: Direct anastomosis of lymph vessels to small veins, with no tissue transfer or excision.	Microsurgical transfer: Transfer of lymph nodes with vasculature from a donor site (commonly the patient's groin, neck, or abdomen) to the affected limb; new vascular channels are created.	(46, 90, 91)
Invasiveness	Minimally invasive; small skin incisions (normally 2–3 cm), often done as outpatient or short-stay procedure; procedure may be done under local or regional anesthesia.	Moderately invasive; some procedures are performed under general anesthesia, some procedures involve harvesting lymph nodes from a donor site (e.g., donor sites may include the groin or abdomen), and often involve multiple days hospitalization.	(90, 91)
Ideal lymphedema stage	Most effective in early stage BCRL (ISL Stage I–II); especially successful when functional lymphatic channels remain; not as successful in advanced fibrotic disease with obliterated lymphatics.	Effective in a wider stage range of BCRL (ISL Stage II–III); especially useful when few or no functioning lymphatics remains; even for patients with advanced fibrosis.	(46, 47)
Onset of improvement	Fast improvement typically occurs within a few weeks to 3 months; initial effect maximized early and likely plateauing thereafter.	Typically slower and gradual improvement over 12–18 months; possibly a second peak of improvement as transferred nodes fully mature.	(47)
Volume reduction	∼30%–50% average reduction in excess limb volume; quick drainage of fluid; limited effect on established fatty or fibrotic tissues.	∼30%–40% reduction in excess limb volume; gradual decrease in fluid with potential to decrease the fibrotic or fatty tissues over time.	(47, 57, 92)
Infection reduction	Significantly reduces recurrence of cellulitis; reports of 0% postoperative cellulitis in some studies; large decrease in emergency department visits for fluctuating episodes.	Reduces infection risk over time; while not often quantified, improved lymphatic flow after VLNT is correlated with fewer infections.	(57, 59, 62, 65, 93)
Durability	Good durability, but early occlusion of 20%–40% of anastomoses within the first year may limit long-term effect; those that remain patent are likely to be durable.	Possibly higher long-term durability; transferred lymph nodes facilitate lymphangiogenesis and create stable alternative drainage pathways over time, with improvements continuing beyond 18–24 months.	(47)
Complication rates	Minimal; rare incidence of minor complications such as skin irritations or lymph leak incidents (<5%); no major surgical complications reported.	Moderate; donor site complications (∼12%); recipient site complications (<5%) include seromas, infections, or flap failure (∼7%).	(46)
Other considerations	Requires advanced training in supermicrosurgery; repeatable procedure; donor site morbidity is minimal because no tissue is harvested.	Requires microsurgery expertise; potential for donor site morbidity due to node harvesting; ability to be combined with breast reconstruction procedure [e.g., deep inferior epigastric perforator (DIEP) flap].	(75, 91)

LVA and VLNT are not mutually exclusive (74) and may be performed together or at separate times, often referred to as “combined physiologic reconstruction” (75). For example, a patient who has moderate BCRL may have LVA immediately for rapid reduction of fluid and have a VLNT surgery a year later to sustain drainage and manage residual edema.

A brief summary of reported complication rates for LVA and VLNT is presented in Table 3. The comparison shows why LVA is generally considered the first surgical option, since there is essentially no procedure-related risk. Furthermore, when LVA is successful, it can provide substantial improvement for the patient, but if it is unsuccessful, then VLNT or liposuction can still be considered while maintaining future surgical options for managing BCRL.

**Table 3:** Summary of complication rates—LVA vs. VLNT in BCRL surgical care.

Procedure	Reported complication rate	Common complications	References
LVA (lymphovenous anastomosis)	∼0%–5% minor complications. No major complications.	Minor wound infections, mild lymphatic leakage at incision sites, or anastomotic failure/occlusion. No donor site morbidity.	(46, 59)
VLNT (vascularized lymph node transfer)	∼15%–20% total complication rate (donor and recipient sites combined). ∼12% donor site complications, ∼7% recipient site. Most complications are minor.	Donor site: seroma, infection; rare donor limb lymphedema (0%–5%). Recipient site: infection, dehiscence, rare flap thrombosis (≤5%)	(47)
Debulking (e.g., SAPL or Charles)	Higher morbidity; complication rates vary by technique and center	SAPL: transient paresthesia, bruising, bleeding, and possible transfusion. Charles: graft loss, infection, chronic wounds, distal edema. High dependence on compression	(63)

SAPL, suction-assisted protein lipectomy or liposuction for lymphedema.

## Future directions in surgical lymphedema care

Some of the promising and important developments include the following:

### Preventive (prophylactic) lymphatic surgery

A key advancement has been the development of strategies to prevent BCRL during initial treatment for breast cancer. The Lymphatic Microsurgical Preventive Healing Approach (LYMPHA) consists of performing an LVA at the time of ALND between a transected arm lymphatic and a nearby axillary vein (76). This functions as a simultaneous preventive lymphatic reconstruction.

For example, in a study by Boccardo et al., 74 breast cancer patients with a plan to undergo axillary dissection underwent LYMPHA. After 4 years of follow-up, only 3 patients (4.05%) developed BCRL. Lymphoscintigraphy also showed the anastomoses remained patent in the long term, with incidence rates much lower than the typically reported incidence of lymphedema following conventional axillary dissection (13%–65%) (76).

Other follow-up studies and a meta-analysis found similar reductions in incidence of lymphedema following immediate lymphatic reconstruction at the time of nodal dissection (77–79). As more breast surgeons and microsurgeons work together, it is likely that prophylactic LVA will be commonplace and incorporated as part of standard surgical care for high-risk patients undergoing mastectomy or nodal surgery.

However, broader implementation of this approach is somewhat hindered by the requirement for advanced microsurgical training and the practical challenges of performing LVA rapidly, while incorporating into oncologic procedures without affecting oncologic outcomes. Current data support its oncologic safety (80).

### Refinements in imaging and mapping

Recent developments have led to new configurations of lymphatic-venous anastomosis to improve long-term patency and flow. One new approach is the “octopus” LVA, which connects a single vein to multiple lymphatic vessels (81). Another is the “flow-through” venous flap, which uses a vein graft to connect multiple lymphatic channels with a distal lymphatic outlet (82). The third is “overlap” or “in-parallel” LVA, which allows bidirectional flow and may promote long-term stability of the anastomosis (83). Although the ideal configuration remains debatable, they are all intended to improve long-term anastomotic function and lymphatic drainage.

### Advanced microsurgical techniques

Advances in medical imaging continue to improve the accuracy and effectiveness of lymphatic surgery. Magnetic resonance lymphangiography (MRL) provides three-dimensional visualization of lymphatic anatomy while greatly improving preoperative planning for LVA and VLNT by detailing potentially viable lymphatic channels and their course (84–86).

Intraoperative visualization is also advancing through more compact near-infrared fluorescence systems for indocyanine green (ICG), which improve real-time assessment of lymphatic flow during LVA procedures (36). These imaging systems can improve intraoperative accuracy and allow more objective postoperative monitoring and timely reintervention (36, 85).

### Robotic supermicrosurgery

Robotic assistance is being explored as a tool for addressing the technical limitations of human precision in supermicrosurgical procedures. The introduction of purpose-built microsurgical robots (such as the MUSA system) has led to the first-in-human clinical trials and studies for LVA (66, 87).

These systems can filter tremor and scale hand movements to the micron level, and they can help make LVA procedures more consistent and enhance the precision of microvascular suturing (88). In the long term, robotic systems may broaden availability by permitting safer LVA, remote microsurgery, and even partial automation of the anastomosis (89).

LVA marks a transition from solely palliation toward actual restoration of lymphatic function. Although there are remaining challenges, predominantly with early identification of patients who are good candidates for LVA, or verifying the outcomes in rigorous clinical studies, each of these emerging areas of focused development is promising.

Further advances in robotic systems, biologic therapies, and growth factor applications will help improve outcomes moving forward.

## Conclusion

Lymphovenous anastomosis represents a major advance in the treatment of breast cancer-related lymphedema, shifting away from merely symptomatic treatment, to the restoration of lymphatic flow physiologically. Recent prospective trials and meta-analyses support promising evidence of safety and a clinically meaningful change in limb function, quality of life, and infection rates, particularly in early-stage disease. Although standardized long-term outcome data are still lacking, improvements in imaging and robotic microsurgery, as well as preventative treatment such as LYMPHA, are improving both precision and availability to training. The future of lymphedema surgery relies upon early identification of patients, multidisciplinary collaboration in care, and rigorous reporting on patient outcomes, to realize the full potential of LVA as a part of rethinking physiologic reconstruction in the modern era.
